# Endophytic Fungi and Ecological Fitness of Chestnuts

**DOI:** 10.3390/plants10030542

**Published:** 2021-03-13

**Authors:** Rosario Nicoletti, Gabriele Loris Beccaro, Agnieszka Sekara, Chiara Cirillo, Claudio Di Vaio

**Affiliations:** 1 Research Center for Olive, Fruit and Citrus Crops, Council for Agricultural Research and Economics, 81100 Caserta, Italy; rosario.nicoletti@crea.gov.it; 2 Department of Agricultural Sciences, University of Naples Federico II, 80055 Portici, Italy; claudio.divaio@unina.it; 3 Department of Agricultural, Forest and Food Sciences, University of Turin, 10095 Grugliasco, Italy; gabriele.beccaro@unito.it; 4 Department of Horticulture, University of Agriculture, 31-425 Krakow, Poland; agnieszka.sekara@urk.edu.pl

**Keywords:** *Castanea* spp., defensive mutualism, endophytes, mycorrhizae, plant growth promotion

## Abstract

Chestnuts (*Castanea* spp.) are plants of relevant economic interest in the agro-sylvicultural contexts of mountain regions throughout the temperate zone, particularly in the northern hemisphere. In recent years, several biological adversities have repeatedly endangered species belonging to this genus, calling for coordinated actions addressed to contrast their decline. These actions have mainly focused on the control of key pests/pathogens and the improvement of resistance/tolerance by the plant host, while the role of microorganisms as mediators of interactions between plants and the noxious agents has been less considered, essentially by reason of a limited knowledge on their ecological impact. In line with the increasing awareness of the basic importance of microbial symbionts in regulating plant fitness in both natural and crop contexts, this paper offers an overview on the occurrence and effects of endophytic fungi of chestnuts.

## 1. Introduction

Chestnuts (*Castanea* spp.) are geographically distributed in three main areas throughout the world (Asia, Europe, North America) where they have an invaluable cultural heritage but also an important economic and environmental role in many agroforestry systems. Their nuts provided for centuries a dietary staple in rural areas and, when dried, a stored food for the whole year; the wood is still today used as firewood or building timber.

The genus *Castanea* (*x* = 12, 2*n* = 24), chestnuts and chinkapins, belongs to the Fagaceae (Cupuliferae) family, which includes 6 genera (*Castanea*, *Quercus, Castanopsis*, *Fagus*, *Nothofagus, Lithocarpus*) and approximately 1000 species. It is closely related to the genus *Castanopsis* [1], is widespread in the boreal hemisphere and encompasses 13 species. The natural distribution of the European or sweet chestnut (*C. sativa*) covers Europe and some Mediterranean countries. In China, Japan, Vietnam, North and South Korea *C. mollissima*, *C. crenata*, *C. seguinii*, *C. henryi* occur. In North America, *C. dentata* is still present only along the Appalachian Mountains, due to the devastating impact of the chestnut blight. *Castanea pumila* is found in the southeastern United States [2,3].

*Castanea* species show different ecological and morphological traits, vegetative habit, wood and nut characteristics, pedoclimatic adaptability, resilience and resistance to biotic and abiotic stresses, reflecting the adaptation of this genus to different environmental conditions [4]. The main cultivated species are *C. sativa*, *C. mollissima* and *C. crenata,* due to their large nut size, and marroni cultivars (*C. sativa*) are considered the most commercially valuable. *Castanea sativa* and *C. dentata* are also cultivated for timber production, whilst many interspecific hybrids are used for nut production or as rootstocks [5].

Chestnut cultivation has been affected by several problems that brought to a dramatic collapse of the whole production system for many years, particularly in Europe and North America. Most of the mountain populations in the European countries were dependent on chestnut farming, both for timber and for fruits (over 1 mln tons of nuts per year at the beginning of the 20th century) [6]. The progressive introduction of new pests and diseases caused massive damages to *C. sativa* and *C. dentata* cultivations. Moreover, the industrial era and the introduction of more remunerative crops (potatoes, cereals) caused the abandonment of large chestnut growing areas. However, over the past 15 years the global world chestnut production has been growing even with some fluctuations, mainly due to Chinese production increase, reaching approximately 2.3 mln tons of fresh fruits in 2019 over more than 600,000 hectares. Eastern Asia and Mediterranean Europe are the two main production areas supplying, respectively, almost 90% and over 7% of the chestnuts produced worldwide. The intense scientific and agronomic activity promoted in the last decades of the 20th century by the Chinese government provided strong tools for improving chestnut cultivation. The increase in global chestnut production was driven both by the Chinese policies and by the global population growth and popularity of healthy eating. These key drivers are expected to continue promoting growth of the chestnut crop in the future [3].

## 2. Ecological and Technical Features of Chestnut Orchards

The ecological features of chestnut orchards, their agronomic management and architecture strongly depend on species and country. The best soils for chestnut are deep, soft, volcanic in origin, and rich in phosphorus, potassium, and organic matter. The soil pH should be in the range 5.0−6.5; therefore, soils with active limestone are not optimal, because *Castanea* species are very sensitive to high pH values. Soil permeability is very important. In fact, the crop performs better in well-drained, loam to sandy-loam soils, while heavy, washed out, clayey, stagnant soils which favor root rot must be avoided [6].

Chestnut tolerates cold winters and requires average temperature of 8–15 °C, with a monthly average of 10 °C for at least six months. *Castanea sativa* is more cold-resistant (−15 to −20 °C) than the Euro-Japanese hybrids. Despite late bud-break (March–April in the Northern Hemisphere), the trees may be prone to spring frosts which damage young shoots. Temperatures of 27−30 °C are necessary during pollination. European and Japanese cultivars require about 800–900 mm/year of rainfall, well distributed during the growing season, while Euro-Japanese hybrids and *C. mollissima* are much more waterdemanding (1200–1300 mm/year). In temperate climates, sweet chestnut should not be planted above 800–900 m, while for hybrids the maximum altitude is about 500–600 m [5].

Ecological features of a chestnut orchard can be influenced by many natural and anthropogenic parameters, such as soil, climate, rootstock, cultivar, cover crops, irrigation system and fertilization [7]. Concerning soil, an increase in the organic matter content is often obtained through sheep and cattle pasture, outside the harvesting period, or with the use of manure (10–15 tons/ha/year). Mown grass, leaves, husks and the small pruned material are often left on the ground as they provide further enrichment in organic matter. Fertilization of the orchard is every year carried out according to soil conditions and uptakes [8]. Biological or conventional management are both adopted by chestnut growers and could strongly influence the crop ecological features.

The orchard model, traditional or intensive, could also strongly influence the crop ecological features. Traditional orchards are mainly located in mountainous areas while intensive orchards are a relatively recent practice and are usually located in lowlands, quite far from the typical ecological conditions of the species [9]. For all species, except in traditional European chestnut orchards, the general trend is to increase plant density to develop in a relatively short time maximum bearing per unit area. Plantation density can affect the orchard microclimatic conditions and can range from 100 to 550 trees/ha, based on species, variety, genotype-environment interactions and cultural practices. For traditional plantations of *C. sativa*, spacing ranges from 8–10 m apart in rows and 8–12 m between rows. For the most vigorous Euro-Japanese hybrids the distances range between 7 × 8 m (178 trees/ha) and 8 × 10 m (125 trees/ha). *Castanea sativa* and the Euro-Japanese hybrids can be cultivated in high density plantations (3 m × 10 m). For *C. crenata* distances of about 5 × 7 m (285 trees/ha, in deep fertile soils) or 7 × 7 m (204 trees/ha) are recommended. *Castanea mollissima*, grown in China and the Unites States, is managed in a high or semi-high density scheme, due to the smaller tree size. Planting patterns may be rectangular, squared or triangular, but the first scheme is mostly used because of easier management [3]. Nowadays, many traditional plantations need renewal and recovery after years of abandonment or following the attacks of pests and diseases that have compromised their efficiency.

## 3. The Relevance of Microorganisms in the Management of Chestnuts

Besides climatic and agronomic aspects, fitness and productivity of chestnuts are remarkably influenced by the manifold interactions established with different kinds of microorganisms. As in most forest trees which are commercially exploited in both natural and anthropized contexts, members of this component of biodiversity occur systematically and play several functional roles on both the roots and the above-ground plant parts. It can be said that they ultimately produce notable direct and indirect effects on the economy of the resident communities in the areas where chestnuts shape ecosystems, landscape and farming activities.

Fungi undoubtedly represent the main group of microbes associated with chestnut plants, variously influencing their fitness. A basically topographic difference separates species associated with roots, many of which pertain to the well-known category of the mycorrhizae. Ectomycorrhizal fungi (EMF) are well-documented as symbionts of *Castanea* spp. [10], abundantly covering the smallest branchings of a root system which is strong, expanded and penetrates the soil deeply. EMF provide incontestable beneficial effects to the chestnut orchard ecosystem. In fact, they stimulate release of nutrients from the soil sorption complex, making them available to the host plant. The constant access to water and mineral resources makes trees more resilient to biotic and abiotic stresses. Indeed, the biochemical cross-talk between fungi and host plants shapes the relationship and increases fitness of both symbionts. Reactive oxygen species may be essential initial products for regulating interactions during the early stages of EMF establishment and signalling molecules for symbiosis establishment. In this regard, oxidative bursts were observed two hours after inoculation of the model EMF *Pisolithus tinctorius* on *C. sativa* roots, followed by increased superoxide dismutase and catalase activities in root cells [11].

EMF change in composition with the age of the trees, shaping ecological succession. Mature trees can host up to 46 EMF species [12], while 39 EMF genera were collected from root tips of 100-year-old chestnut [13]. The young chestnut trees are hosts of “earlystage” EMF, such as *Scleroderma* spp., *Laccaria* spp. and *Cenococcum geophilum*. The easy dispersal and fast colonization capacity by these fungi on young root systems, even in stressful ecological conditions, can be suitable for nursery inoculation [14,15]. Late-stage EMF, generally dominant in mature stands, comprise *Amanita*, *Boletus*, *Cantharellus, Cortinarius*, *Lactarius*, *Russula*, and *Tricholoma* species, which are of particular interest as valuable culinary ingredients and can provide additional profits to local communities [16]. The Russulaceae members are among the EMF associated with the residual natural stands of *C. dentata* in North American forests [17]. Moreover, successful trials have been carried out demonstrating the ability by truffle species (*Tuber aestivum*, *T. uncinatum*, and *T. brumale*) to colonize roots of *C. sativa*. The development of commercial inoculation protocols with these species is of high economic relevance [18].

Hypogeous EMF surveys provide valuable information on the reproductive investment by chestnut-related symbiotic fungi. However, these trials partially reflect the soil fungal diversity and interspecies relations in the chestnut rhizosphere [19]. The cited study showed approximately 35% discontinuity in the fungal species composition between the above and below ground methods of sampling of EMF in *C. dentata*. Of the 46 sequences identified on root tips, only 16 represented the above ground mushroom survey. Among the EMF identified molecularly from root tips of *Castanea* × *coudercii* [20], 13 species had not been previously documented as symbionts of chestnuts, including *Hymenogaster* sp., *Thelephora* sp., *Tomentella* sp.

In the early 20th century, research on EMF of *C. sativa* and *C. dentata* was highly disturbed by large-scale mortality of trees due to the spread of fungal diseases [21]. Both American and sweet chestnut represent the main components of the respective forest ecosystems, shaping their ecological associations. Considering that their extinction could have triggered widespread environmental disturbances, an extensive reintroduction project was realized involving disease resistant hybrids of *C. sativa* with *C. crenata* and *C. mollissima*, or plants grafted on hybrid rootstocks [22]. Hybrid and grafted chestnuts, which exhibit better performance, could develop individual EMF associations, diversity and colonization rates. In this respect, a previously cited study [20] determined the presence of 9 orders, 15 families, 19 genera and 27 species of EMF fungi on *Castanea* × *coudercii*, most of them generalist, early-stage species. *Scleroderma* spp. were the most abundant, while *C. geophilum* was found in most of the trees but colonized a small fraction of root tips. Although the authors did not confirm differences in EMF diversity between grafted and non-grafted specimens, the results could concurrently improve the management of chestnut agro-ecosystems and increase the production of mushrooms. In another study *Castanea* hybrids had similar interactions with soil biota to their parent species, *C. dentata*, and may fill a similar below-ground niche [23]. However, non-native pathogen presence in restoration sites can affect growth and survival of *Castanea* hybrids. Colonization by *Cortinarius* spp. facilitated the survival of native *C. dentata* infected by *Cryphonectria* sp. [24], and inoculation with *Hebeloma crustuliniforme* decreased disease symptoms in *C. sativa* infected by *Phytophthora cambivora* [25]. The cited research highlights that natural mutualistic interactions may be effective in the control of chestnut pathogens.

## 4. Occurrence of Endophytic Fungi in Chestnuts and Ecological Implications

Although characterized by synchronized development inside the plant tissues and nutrient transfer at the interfaces [26], EMF are not included in the category of endophytes since at some extent they also grow saprophytically in the soil. Conversely, the definition of endophytes, on which this paper is more specifically centered, does not reflect a specialized nutritional function, and it is conventionally applied to microorganisms that colonize living, internal tissues of plants without causing any immediate, overt negative effect [27].

Data concerning occurrence of endophytic fungi of chestnuts (Table 1) are only available from a low number of countries, indicating that consideration for the ecological implications and the economic impact related to this component of biodiversity is still quite limited. Possibly connected with a higher importance as an economic crop in Europe, most investigations have been carried out on *C. sativa*, with 76 taxa reported so far (2/3 of which identified at the species level), while the species *C. crenata, C. dentata* and *C. mollissima* appear to have been less frequently investigated, basically in Japan, the United States and China. As a general aspect, identifications concerning stem (shoots, branches, etc.) refer to the occurrence of endophytes in subcortical tissues, while no findings were reported from xylem [28,29].

In some cases, conditions of detection did not fully meet the basic requirements referring to the true endophytic condition. In fact, a recent study based on ITS meta-barcoding was addressed to establish whether fungal communities within cynipid galls are different from foliar endophytes. Unfortunately, results were basically presented with reference to classes to which the detected OTUs belong, and identifications at the species level was only carried out for the main OTUs, without distinguishing their origin (gall or leaf) [30]. Therefore, entries in Table 1 referring to this study must be taken with circumspection. Moreover, some uncertainty is entailed in a few reports concerning endophytic fungi associated with cankers produced by *C. parasitica* on both *C. sativa* and *C. dentata* [31,32,33,34,35,36]. Even if in these cases the plant material used for isolations was not asymptomatic following infection by a known pathogen, the sterilization procedure ensured that at least the isolated fungi had colonized the plant tissues before sampling and were not epiphytic contaminants.

**Table 1:** Endophytic fungi reported from *Castanea* spp.

Endophyte ^1^	Plant Part	Country	Reference
*Castanea sativa*			
*Acremonium* cf. *curvulum*	shoot (phellem)	Bellinzona, Switzerland	[28]
*Alternaria alternata*	several plant parts	Eurobin and Monbulk, Australia	[37]
	shoot	Ticino, Switzerland	[38]
	bud	Ankara, Turkey	[39]
	bark	Vinhais, Portugal	[40]
*Alternaria* sp.	shoot	Geneve and Ticino, Switzerland	[41]
	leaf or gall	Southern Tuscany, Italy	[30]
	bud	Ankara, Turkey	[39]
*Apiognomonia errabunda*	leaf	Cureglia and Zarei, Switzerland	[42]
Arcopilus aureus *	bark	Vinhais, Portugal	[40]
*Arthrinium arundinis*	leaf	Vejoris, Spain	[43]
*Aspergillus* sp.	stem	Black Sea region, Turkey	[31]
	bud	Ankara, Turkey	[39]
*Asterosporium* sp.	shoot (phellem)	Bellinzona, Switzerland	[28]
*Aureobasidium pullulans*	shoot	Ticino, Switzerland	[38,41]
*Aureobasidium* sp.	bud	Ankara, Turkey	[39]
*Biscogniauxia mediterranea*	bark	Valpaços and Vinhais, Portugal	[40]
*Botryosphaeria dothidea*	shoot	Ticino, Switzerland	[41]
	leaf or gall	Southern Tuscany, Italy	[30]
*Botryosphaeria* sp.	several plant parts	Eurobin and Monbulk, Australia	[37]
*Botryotinia pelargonii*	leaf or gall	Southern Tuscany, Italy	[30]
*Botrytis cinerea*	several plant parts	Eurobin and Monbulk, Australia	[37]
*Chaetomium* sp.	several plant parts	Eurobin and Monbulk, Australia	[37]
*Cladosporium cladosporioides*	several plant parts	Eurobin and Monbulk, Australia	[37]
*Cladosporium* sp.	fruit	Eurobin and Monbulk, Australia	[37]
	bud	Ankara, Turkey	[39]
*Colletotrichum acutatum*	shoot (phellem)	Bellinzona, Switzerland	[28]
	leaf, shoot	Monti Cimini, Italy	[44]
	leaf	Vejoris, Spain	[43]
*Coprinellus domesticus*	bark	Oghuz, Azerbaijan	[33]
*Coryneum modonium*	shoot (phellem)	Bellinzona and Murg, Switzerland	[28]
*Cryphonectria parasitica*	shoot (phellem)	Bellinzona, Switzerland	[28]
	sprout	Fossemagne, France	[45]
	bark	Valpaços and Vinhais, Portugal	[40]
*Cytospora chrysosperma*	bark	Valpaços, Portugal	[40]
*Cytospora diatyrpelloidea*	bark	Valpaços and Vinhais, Portugal	[40]
*Cytospora eucalypticola*	bark	Valpaços, Portugal	[40]
*Cytospora quercicola*	bark	Vinhais, Portugal	[40]
Dendrostoma castaneum *	shoot (phellem)	Bellinzona and Murg, Switzerland	[28]
	fruit	Toricella, Switzerland	[46]
	branch	Astroni Nature Reserve, Italy	[47]
*Diaporthe amygdali*	bark	Oghuz, Azerbaijan	[33]
*Diaporthe eres*	shoot	Geneve and Ticino, Switzerland	[38,41]
*Diaporthe foeniculina*	branch	Astroni Nature Reserve, Italy	[48]
*Diaporthe* sp.	shoot (phellem)	Bellinzona and Murg, Switzerland	[28]
	leaf or gall	Southern Tuscany, Italy	[30]
	bud	Ankara, Turkey	[39]
*Diplodia seriata*	leaf or gall	Southern Tuscany, Italy	[30]
*Diplodina castaneae * *	shoot (phellem)	Bellinzona and Murg, Switzerland	[28]
	stem, twig	Chablais and Ticino, Switzerland	[48]
	stem	Northern Spain	[48]
	bark, stem	Ismailly, Qabala, Sheki (Azerbaijan)	[33]
*Epicoccum nigrum*	several plant parts	Eurobin and Monbulk, Australia	[37]
	bark	Balakan, Azerbaijan	[33]
	bud	Ankara, Turkey	[39]
*Eutypella* sp.	bark	Zagatala, Azerbaijan	[33]
*Fusarium ciliatum*	leaf or gall	Southern Tuscany, Italy	[30]
*Fusarium lateritium*	leaf or gall	Southern Tuscany, Italy	[30]
*Fusarium oxysporum*	leaf or gall	Southern Tuscany, Italy	[30]
*Fusarium* sp.	fruit	Eurobin and Monbulk, Australia	[37]
	leaf or gall	Southern Tuscany, Italy	[30]
	leaf	Vejoris, Spain	[43]
	bud	Ankara, Turkey	[39]
Gnomoniopsis castaneae *	several plant parts	Eurobin and Monbulk, Australia	[37]
	bark, flower, leaf	several locations in New Zealand	[29]
	several plant parts	Cuneo province, Italy	[49,50]
	flower, leaf, stem	Southern Australia	[51]
	shoot	several locations in Northern Italy	[52]
	fruit	several locations in Switzerland	[53]
	buds	Aosta Valley and Piedmont, Italy	[54]
	shoot	Geneve and Ticino, Switzerland	[38,41]
	several plant parts	Monti Cimini, Italy	[44,55]
	leaf or gall	Southern Tuscany, Italy	[30]
	leaf	Vejoris, Spain	[43]
	leaf	Netherlands	[56]
	branch	Astroni Nature Reserve, Italy	[47]
*Hyphodermella rosae*	bark	Ismailly and Shaki, Azerbaijan	[33]
*Hypoxylon fragiforme*	shoot (phellem)	Bellinzona and Murg, Switzerland	[28]
*Irpex lacteus*	bark	Balakan, Azerbaijan	[33]
*Jattaea* sp.	bark	Zagatala, Azerbaijan	[33]
*Massarina* cf. *quercina*	shoot (phellem)	Murg, Switzerland	[28]
*Mollisia* sp. (= *Cystodendron* sp.)	shoot (phellem)	Bellinzona, Switzerland	[28,57]
*Monodictys castaneae*	shoot (phellem)	Bellinzona, Switzerland	[28]
*Mucor fragilis*	bark	Vinhais, Portugal	[40]
*Neocucurbitaria cava* *	leaf or gall	Southern Tuscany, Italy	[30]
*Neopestalotiopsis* sp.	bark	Asturias, Spain	[35]
*Neopestalotiopsis zimbabwana*	bark	Asturias, Spain	[35]
*Nigrospora* sp.	several plant parts	Eurobin and Monbulk, Australia	[37]
*Ophiovalsa* cf. *suffusa*	shoot (lenticel)	Bellinzona, Switzerland	[28]
*Paraconiothyrium brasiliense*	bark	Vinhais, Portugal	[40]
	branch	Astroni Nature Reserve, Italy	[47]
*Penicillium glabrum*	bark	Vinhais, Portugal	[40]
*Penicillium* sp.	several plant parts	Eurobin and Monbulk, Australia	[37]
	stem	Black Sea region, Turkey	[31]
	bark	Marche, Italy	[34]
	bud	Ankara, Turkey	[39]
	bark	Valpaços, Portugal	[40]
	branch	Astroni Nature Reserve, Italy	[47]
*Pestalotiopsis* sp.	leaf	Vejoris, Spain	[43]
	bark	Asturias, Spain	[35]
	bud	Ankara, Turkey	[39]
*Pezicula cinnamomea*	shoot (phellem)	Bellinzona and Murg, Switzerland	[28]
*Phaeococcus* sp.	shoot (phellem)	Murg, Switzerland	[28]
*Phoma* sp.	shoot (phellem)	Bellinzona, Switzerland	[28]
	several plant parts	Eurobin and Monbulk, Australia	[37]
	bud	Ankara, Turkey	[39]
*Pilidiella castaneicola* *	shoot	Bellinzona and Murg, Switzerland	[28]
*Ramichloridium* sp.	shoot (phellem)	Murg, Switzerland	[28]
*Rhizoctonia* sp.	shoot (phellem)	Bellinzona, Switzerland	[28]
*Rhizopus* sp.	bark	Vinhais, Portugal	[40]
*Sclerotinia pseudotuberosa*	bark, bud, fruit	Viterbo province, Italy	[58]
*Sordaria rabenhorstii* *	bark	Valpaços, Portugal	[40]
*Sordaria* sp.	several plant parts	Eurobin and Monbulk, Australia	[37]
	leaf or gall	Southern Tuscany, Italy	[30]
*Stemphylium vesicarium*	leaf or gall	Southern Tuscany, Italy	[30]
*Trichoderma atroviride*	scion	Ticino, Switzerland	[38]
	leaf	Vejoris, Spain	[43]
*Trichoderma hamatum*	shoot	Ticino, Switzerland	[38]
*Trichoderma koningiopsis*	bark	Shaki, Azerbaijan	[33]
*Trichoderma* sp.	stem	Black Sea region, Turkey	[31]
	bark	Qakh, Azerbaijan	[33]
	bark	Marche, Italy	[34]
*Trichothecium roseum*	leaf or gall	Southern Tuscany, Italy	[30]
*Umbelopsis isabellina*	bark	Balakan and Qabala, Azerbaijan	[33]
	bark	Vinhais, Portugal	[40]
*Xenoacremonium falcatum*	bark	Balakan and Qabala, Azerbaijan	[33]
*Xylaria* sp.	branch	Astroni Nature Reserve, Italy	[47]
*Castanea crenata*			
*Alternaria* sp.	leaf	Kashiwa, Japan	[59]
*Astrocystis* sp.	leaf	Kashiwa, Japan	[59]
*Aureobasidium* sp.	leaf	Kashiwa, Japan	[59]
*Botryosphaeria dothidea*	leaf	Kashiwa, Japan	[59]
*Colletotrichum acutatum*	leaf	Kashiwa, Japan	[59]
*Colletotrichum gloeosporioides*	leaf	Kashiwa, Japan	[59]
*Diaporthe* sp.	leaf	Kashiwa, Japan	[59]
*Discula* sp.	leaf	Kashiwa, Japan	[59]
*Glomerella* sp.	leaf	Kashiwa, Japan	[59]
Gnomoniopsis castaneae *	bark, flower, leaf	several locations in New Zealand	[29]
*Induratia fengyangensis* *	leaf	Kashiwa, Japan	[59]
*Nigrospora* sp.	leaf	Kashiwa, Japan	[59]
*Pestalotiopsis* sp.	leaf	Kashiwa, Japan	[59]
*Phyllosticta capitalensis*	leaf	Kashiwa, Japan	[59]
*Xylaria* sp.	leaf	Kashiwa, Japan	[59]
*Castanea dentata*			
*Acremonium implicatum* *	stem	Michigan and Wisconsin, USA	[36]
*Alternaria alternata*	stem	Michigan and Wisconsin, USA	[36]
*Alternaria brassicae*	stem	Michigan and Wisconsin, USA	[36]
*Aspergillus tubingensis*	stem	Michigan, USA	[60]
*Biscogniauxia* aff. *mediterranea*	stem	Michigan and North Carolina, USA	[60]
*Botryosphaeria* sp.	stem	Michigan and Wisconsin, USA	[32,36]
*Daldinia* aff. *childiae*	stem	Michigan, USA	[60]
*Didimostylbe* sp.	stem	Wisconsin, USA	[32]
*Diplodia corticola*	stem	Michigan and Wisconsin, USA	[36]
*Diplodia seriata*	stem	Michigan and Wisconsin, USA	[36]
*Dothiorella* sp.	stem	Wisconsin, USA	[32]
*Epicoccum nigrum*	stem	Wisconsin, USA	[32]
	stem	Michigan, USA	[60]
	stem	Michigan and Wisconsin, USA	[61]
*Fusarium* sp.	stem	Massachusetts, USA	[62]
Gnomoniopsis castaneae *	flower, leaf	Ohaupo, New Zealand	[29]
	stem	Michigan and Wisconsin, USA	[36]
*Mucor circinelloides*	stem	Michigan and Wisconsin, USA	[36]
*Mucor fragilis*	stem	Michigan and Wisconsin, USA	[36]
*Nectria cinnabarina*	stem	Michigan and Wisconsin, USA	[36]
*Nigrospora* aff. *oryzae*	stem	Michigan, USA	[60]
*Paraconiothyrium* sp.	stem	Wisconsin, USA	[32]
*Penicillium glabrum*	stem	Michigan and Wisconsin, USA	[32,36]
*Penicillium spinulosum*	stem	Michigan and Wisconsin, USA	[32,36]
*Pestalotia* sp.	stem	Wisconsin, USA	[31]
*Pestalotiopsis* sp.	stem	North Carolina, USA	[60]
*Pezicula cinnamomea*	stem	Michigan and Wisconsin, USA	[36]
*Pezicula ericae*	stem	Michigan and Wisconsin, USA	[36]
*Pezicula sporulosa*	stem	North Carolina, USA	[60]
*Strasseria* sp.	stem	Michigan and Wisconsin, USA	[36]
*Trichoderma atroviride*	stem	Michigan and Wisconsin, USA	[32,36]
*Trichoderma aureoviride*	stem	Wisconsin, USA	[32]
*Trichoderma citrinoviride*	stem	Michigan and Wisconsin, USA	[36]
*Trichoderma harzianum*	stem	Michigan and Wisconsin, USA	[36]
*Trichoderma* sp.	stem	Massachusetts, USA	[62]
*Tubakia suttoniana* *	stem	North Carolina, USA	[59]
*Umbelopsis isabellina*	stem	Michigan and Wisconsin, USA	[32,36,61]
*Castanea mollissima*			
*Alternaria eichhorniae*	leaf	Qing Long, China	[63]
*Alternaria* sp.	leaf	Qing Long, China	[63]
*Auriculibuller fuscus* *	leaf	Qing Long, China	[63]
*Cercospora canescens*	twig	Qing Long, China	[63]
*Cercospora* sp.	twig	Qing Long, China	[63]
*Colacogloea* sp.	leaf	Qing Long, China	[63]
*Colacogloea terpenoidalis*	leaf	Qing Long, China	[63]
Gnomoniopsis castaneae *	bark, leaf	Ohaupo, New Zealand	[29]
*Kondoa sorbi*	leaf	Qing Long, China	[63]
*Kondoa* sp.	twig	Qing Long, China	[63]
*Papiliotrema* sp.	leaf	Qing Long, China	[63]
*Phlebia acerina*	-	China	[64]
*Sporobolomyces roseus*	twig	Qing Long, China	[63]

^1^ Underlined species are also known as disease agents of chestnuts. * Names with an asterisk are the latest accepted for these species, which are different from the ones used in the corresponding references.

### 4.1. Endophytic Fungi as Plant Disease Agents

Chestnut is affected by many fungal pathogens, which sometimes have caused epidemics endangering this plant in more or less extended geographic contexts. Particularly, the blight and the ink diseases, respectively, caused by *Cryphonectria parasitica* and Phytophthora cinnamomi or *P. cambivora*, have required large-scale management with the concomitant employment of breeding, agronomic and biological control measures [65,66,67,68,69]. As known for many other plant pathogens, these fungi may present a latent stage during the disease cycle which can imply their possible recovery from asymptomatic tissues. In fact, recent investigations on microbial symbionts of several plant species are disclosing cases where fungal pathogens may persist in the endophytic condition for prolonged periods [70,71,72], supporting the hypothesis that at least some strains could behave as true endophytes in the absence of factors stimulating their pathogenicity. In the case of *C. sativa*, this aspect has been proposed as a possible explanation for the recovery of *C. parasitica* from asymptomatic coppice shoots of *C. sativa* in Switzerland [28]. This finding was followed by another report from France where the fungus could be re-isolated from asymptomatic tissues for up to seven months after artificial inoculation [45]. Although molecular methods have been developed for the detection of *C. parasitica* in plant tissues by real-time PCR [73,74], to the best of our knowledge no systematic investigations have been dedicated to the eventual endophytic spread of this fungus.

The occasional endophytic occurrence of the chestnut blight agent must not be confused with the well documented case of hypovirulent strains. In fact, although they do not cause major damage to the plants, the concept of hypovirulence basically reflects the establishment of a pathogenic interaction [69]. On the other hand, it is also to be considered that hypovirulent strains can be recovered from healed cankers in plants where they had been experimentally inoculated, even at a certain distance from the previously symptomatic tissues [40]. Considering that the family Cryphonectriaceae mainly includes species with an endophytic lifestyle [75,76], the spread of hypovirulent strains within chestnuts, either after inoculation or in consequence of natural dispersal represents a case study deserving to be thoroughly analysed in view of a more formal assessment of how considering these border line ecological interactions. Indeed, recent observations seem to support the conjecture that mycoviruses can directly interact with the genome of fungi and convert necrotrophic pathogens into unharmful or even beneficial endophytes [77,78]. Moreover, additional data have pointed out that the pathogenic transition in *C. parasitica* was probably driven by the loss of genes involved in carbohydrate metabolism [79].

The additional fungal species known as pathogens of chestnuts that have been reported to occur as endophytes are underlined in Table 1. Some species are occasionally reported to cause dieback and cankers, such as *Coryneum modonium* (= *Melanconia modonia*) [80], *Diplodina castaneae* (= *Sirococcus castaneae*), better known as the agent of the Javart disease of chestnut [48], *and Dendrostoma castaneum* [28,46]. The latter represents a new name for *Amphiporthe castanea* (Diaporthales, Erythogloeaceae) [81], known to be widespread in Europe on *C. sativa*; this species is probably vertically transmitted as it has been found to infect seeds before harvest [46]. The recent description of several *additional Dendrostoma* species associated with *Castanea* spp. in Europe and China [81,82] calls for further assessments of the real nature of these symbiotic relationships. *Botryosphaeria dothidea* has been found to cause stem cankers and black rot of nuts on *C. crenata*, respectively, in Korea [83] and Japan [84,85]; moreover, on *C. sativa* it has been reported from cankers in the Black Sea region of Turkey [31] and from rotted nuts in Croatia, along with *Diaporthe eres* [86]. In China, the latter is also known to cause a brown margin leaf blight of *C. mollissima* [87]. Other fungi which damage fruits are *Sclerotinia pseudotuberosa* (= *Ciboria batschiana*), causing brown rot of nuts [52], *Colletotrichum acutatum* reported as the agent of a pink rot [88], *Trichothecium roseum*, *Fusarium oxysporum*, *Botrytis cinerea*, species of *Penicillium*, *Aspergillus* and *Mucor* and the above cited *D. castaneum*, whose incidence in determining moldy nuts is basically a secondary effect to erosions caused by moth and weevil larvae [46,89]. Conversely, so far there are no reports concerning the endophytic occurrence of the leaf spot agent *Mycosphaerella maculiformis* [44] or *Fistulina hepatica* which causes discoloration and red stain of chestnut wood [90].

Last but not the least, *Gnomoniopsis castaneae* has recently become the hottest fungal associate of chestnuts, being able to establish various types of interaction. This species was typified by Visentin et al. [50] quite recently, after having been described as *Gnomonia pascoe* (= *Discula pascoe*) from chestnut standings near Cuneo (Italy), displaying an endophytic behavior in several plant parts but causing disease symptoms on ripened fruits [49]. It now seems quite clear that this species was already known in taxonomy since 1879 with the name *Phoma endogena* [91]; later on, several independent reports used the names *Phomopsis endogena, Phomopsis castanea* and *Phomopsis viterbensis*, which are now considered as synonyms based on pictures and descriptions, even if a direct comparison of cultures is no more possible [52]. As previously mentioned for *D. castaneum*, the species is reported to be vertically transmitted [37]. Although sometimes the fungus was also associated to cankers, most of the old reports concern infection of nuts. Moreover, it has been described as agent of leaf and shoot blight and more recently reported to cause twig canker in Europe and India [41,92].

### 4.2. Endophytic Fungi as Mutualists

Besides having a direct effect on the pathogen, hypovirulent strains of *C. parasitica* can influence the course of chestnut blight by interfering with the development of endophytes. In this respect, an investigation carried out in Portugal showed that, with the single exception of *Penicillium glabrum*, endophytic fungi were less abundant in and around healed cankers after the treatment with hypovirulent strains [40]. Interactions between *C. parasitica* and endophytic fungi are important and deserve to be investigated more in depth. In fact, it has been inferred that, as a general feature, the impact of their antagonistic properties is higher against the hypovirulent than the virulent strains, which implies that they could impair the effectiveness of biological control of chestnut blights by negatively interfering with the natural spread of the former [36,40].

In addition to some species also known as pathogens, such as *G. castaneae* and *C. acutatum*, with a potential antagonistic role against the blight agent [93], several species in Table 1 are reported to be involved at some extent in plant defense against pest and pathogens, thus representing examples of the ecological symbiotic relationship described as defensive mutualism [94]. This list includes *Epicoccum nigrum*, *Fusarium lateritium* and *Paraconiothyrium brasiliense*, but also species of *Aspergillus*, *Chaetomium*, *Penicillium* and *Pestalotiopsis* are frequently mentioned in investigations concerning this issue, particularly with reference to the production of bioactive compounds [95,96,97,98]. However, in this respect the most effective role is probably played by species of *Trichoderma*, representing the most considered antagonists of plant pathogenic fungi even in terms of practical employment in biological control [99,100]. Several species of this genus have been isolated from both *C. sativa* and *C. dentata*, with some indications of a direct involvement in protection against *C. parasitica*. In particular, *T. atroviride* was reported for endophytic occurrence in healthy leaves of *C. sativa* [43] and found to be abundant in healing cankers on *C. dentata* along with *T. aureoviride* [32]. Antagonistic effects by this species have been documented against *G. castaneae* on grafting scions [38]. Moreover, some trials on chestnut plants demonstrated effectiveness by *Trichoderma* strains in reducing blight symptoms [34,62,101]. Finally, in other studies the evaluation of antagonistic properties against *C. parasitica* has been limited to in vitro assays; effectiveness at some extent was observed for strains of Pezicula cinnamomea [28], *Trichoderma* spp., *Penicillium* sp., *Gnomoniopsis* sp., *E. nigrum* and Umbelopsis isabellina [61], *Penicillium* spp. [34] and *Neopestalotiopsis zimbabwana* [35].

### 4.3. Endophytic Fungi and the Cynipid Gall Wasps

As introduced above, fungi reported to occur in galls caused by the wasp *Dryocosmus kuriphilus* (Hymenoptera: Cynipidae) [102] are not to be considered as endophytes in the strict sense, since they develop in altered plant tissues. Nevertheless, many investigations carried out on this biological adversity have considered the possible role of fungi associated with these insect structures and examined their occurrence as endophytes.

The real nature of interactions between cynipid wasps and fungi is still debated. Systematic associations have been ascertained for other insects forming galls, such as midges belonging to the Asphondyliinae which are constantly associated with the cosmopolitan *B. dothidea* on whatever host plants based on a trophic relationship [103]. In the case of *D. kuriphilus*, indications are not univocal. An investigation carried out in Switzerland showed the existence of a possible link between galls and increased spread of chestnut blight. In fact, abandoned galls were found to be frequently colonized by virulent *C. parasitica* and considered to possibly represent the starting points of new infections; at the same time, the association appeared to be more relevant in stands where the insect was resident since longer time [93]. However, in a dedicated study the blight agent was not found on the body of wasps emerging from galls, which seems to rule out their possible role as vectors. Nevertheless, the finding of some endophytes, such as *G. castaneae*, *C. acutatum* and *E. nigrum*, represents an indication of the ability of wasps to act as their vectors, with possible effects on chestnut fitness [104]. Interestingly, the same species were isolated from galls in a Spanish study [43] and again in the course of the previously mentioned Swiss investigation [93], calling for their role in the tripartite relationships with plant and insects to be investigated more in depth. Besides the three above species, the latter study reports a long series of gall-associated fungi, many of which also known as endophytes of chestnuts (Table 1), including *A. alternata, A. pullulans, B. mediterranea, B. dothidea, B. cinerea, F. oxysporum, H. rosae, N. cava, N. oryzae*, *P. brasiliense*, *P. glabrum*, *T. citrinoviride* and *T. harzianum*.

Another mentioned pathogen/endophyte of chestnut, *D. castaneae*, was diffusely isolated from necrotic cynipid galls from several locations in Azerbaijan and Switzerland [48]. Indeed, gall necrosis is thought to severely affect vitality of *D. kuriphilus*, basically impacting the adults inside the galls. Investigations specifically concerning *G. castaneae* showed an exponential increase in gall necrosis during the season, reaching 75.4% in the mid of July. This process, which is likely to be triggered by resident endophytic inoculum, may result in an efficient control of the cynipid in chestnut stands; nevertheless, the high virulence to fruits is assumed to preclude use of this fungus in biocontrol strategies [55].

However, the issue of the relationships between cynipid wasps and *G. castaneae* remains quite controversial [56]. In fact, in another study the fungus could never be isolated from the insects, suggesting unlikeliness that *D. kuriphilus* may act as a vector of viable inoculum. The fungus was present in 33.8% of the buds before oviposition, while no association was detected between fungal colonization and oviposition. Moreover, the number of emerging adults was significantly higher from galls colonized by *G. castaneae* than from non-colonized ones, indicating a possible fungus/pest synergy. These findings suggest that this symbiotic association is asymmetrically favorable to the pest, and that it is eventually established after oviposition [54].

## 5. Conclusions

As for other crop species, our knowledge of the actual impact of endophytic fungi on chestnut fitness and economic performances is still at a preliminary stage. The extent at which research in the field may result in clearer evidence, and practical applications basically rely on the capacity to attain to more accurate assessments of the species assemblages in the different climatic and phytosanitary conditions. As an example, a recent study demonstrated that the yellow crinkle disease pathogen (*Candidatus*-phytoplasma castaneae) significantly changed the structure of fungal communities in chestnut leaves and twigs [63]. However, this analysis based on taxonomic aggregates higher than the species level has quite a limited significance, considering that higher taxa are inclusive of entities with very different ecological roles and impact. Moreover, another recent investigation on fungal rhizobiomes of chestnuts [105] tried to distinguish the species assortment according to the alleged guilds as inferable from categorization proposed in [106]. Based on our previous considerations, particularly on the possible shift endophyte/pathogen which is documented for many fungal species, such attempts appear to be not so useful for investigations aiming at establishing the ecological role played by a certain species and their eventual exploitation in crop management.

More in depth investigations are also required to better understand the nature of processes leading to the endophyte/pathogen conversion, also with reference to genetic diversity in *Castanea* [107]. Besides the above-mentioned role by mycoviruses, this biological phenomenon may also derive by the ability to produce phytotoxins or other compounds involved in disease induction. No investigations have been carried out so far on the production of secondary metabolites by endophytic fungi of chestnuts, with a single exception concerning a strain of *G. castanea* reported to produce abscisic acid and its diol derivative, which are possibly involved in pathogenicity [108].

## Data Availability

Not applicable.
